# An Internet-Delivered Sexual Assault Resistance Intervention for Undergraduate Women (The IDEA3 Trial): Protocol for a Multisite Randomized Controlled Efficacy Trial

**DOI:** 10.2196/72087

**Published:** 2025-10-07

**Authors:** Sarah M Peitzmeier, Charlene Y Senn, Misha Eliasziw, Katie Edwards, Paula Barata, Leanna J Papp, Karen L Hobden

**Affiliations:** 1 Department of Behavioral and Community Health School of Public Health University of Maryland, College Park College Park, MD United States; 2 Department of Psychology University of Windsor Windsor, ON Canada; 3 School of Medicine Tufts University Medford, MA United States; 4 School of Social Work University of Michigan–Ann Arbor Ann Arbor, MI United States; 5 Department of Psychology University of Guelph Guelph, ON Canada; 6 Department of Population Health Sciences University of Central Florida Orlando, FL United States

**Keywords:** education, intervention, resistance, sexual assault, universities, women

## Abstract

**Background:**

Sexual assault (SA) is a serious problem at universities. It is estimated that 1 in 5 women students will experience SA before they graduate.

**Objective:**

The primary aim of this randomized controlled trial is to test whether a synchronous web-based facilitated adaptation of an efficacious intervention (the Enhanced Assess, Acknowledge, Act [EAAA] program) can reduce the 1-year incidence of rape among first- and second-year undergraduate women. Secondary aims will assess the impact of the Internet-Delivered EAAA (IDEA^3^) on other forms of SA (attempted rape, attempted and completed coercion, and nonconsensual sexual contact). Tertiary aims will evaluate effects of the program on (1) survivor self-blame in the event of an assault, (2) 6 known mediators of the intervention effect to guide revision of IDEA^3^ if necessary, and (3) 2 exploratory outcomes measuring acceptance of sexualized aggression and sexual empowerment.

**Methods:**

In this multisite open-label randomized controlled trial, 1920 diverse women students at 2 Canadian and 4 US universities will be randomly assigned to receive either the 12-hour IDEA^3^ (intervention arm) or standard of care (control arm: 60-minute, web-based, live-facilitated consent workshop). Outcomes are measured at baseline, 1 week, 6 months, and 12 months post intervention. SA will be assessed using the Sexual Experiences Survey-Short Form Version. Survivor self-blame and outcomes known to be mediators of EAAA’s reductions in SA will also be measured.

**Results:**

Funding was received in October 2022. Recruitment began on October 2, 2023, and the first participant was randomized on October 23, 2023. As of August 2025, a total of 683 participants have been enrolled and randomized. Data collection will end in May 2027.

**Conclusions:**

If efficacious, IDEA^3^ will be made available to universities in Canada and the United States. To date, 6 provinces in Canada and the Campus SaVE Act in the United States require institutions to provide SA prevention programming. An evidence-based, cost-effective option that can be delivered remotely via the internet has the potential to become the gold standard. The research, therefore, has the potential to impact women’s health and safety internationally.

**Trial Registration:**

ClinicalTrials.gov NCT06058455, https://clinicaltrials.gov/study/NCT06058455

**International Registered Report Identifier (IRRID):**

DERR1-10.2196/72087

## Introduction

An estimated 1 in 5 undergraduate women experience sexual assault (SA) before graduation, driving depression, anxiety, and negative educational outcomes in survivors [1-3]. Unfortunately, few interventions designed for college students have been proven effective in reducing perpetration or victimization [4]. The Enhanced Assess, Acknowledge, Act (EAAA) SA resistance program is the only intervention proven in a randomized controlled trial (RCT) to reduce SA victimization and sustain these benefits beyond 6 months [4]. Compared with participants in the control arm of the trial, attempted and completed rape was reduced by 50% in female undergraduates who took EAAA, with effects lasting at least 2 years [5]. An implementation effectiveness trial of female-identified students at 5 implementing universities replicated the reductions in completed rape and all mediators for students beyond the first year in a more diverse sample [6]. The program also appears to have effects that generalize to reduce nonsexual intimate partner violence by over 50% for at least 12 months [7].

The EAAA SA resistance program consists of four 3-hour units led by 2 trained near-peer facilitators with a group of up to 20 women [8]. The program uses games, mini-lectures, facilitated discussion, and application and practice activities to facilitate knowledge and skill acquisition. Unit 1 (assess) focuses on improving women’s assessment of risk for SA involving male acquaintances and developing problem-solving strategies to reduce perpetrator advantages. Unit 2 (acknowledge) assists women in acknowledging the inherent danger in situations that have become coercive and exploring ways to overcome emotional barriers to resisting known men. Unit 3 (act) offers instruction and practice of various effective resistance options, focusing on common acquaintance SA situations. It also includes 2 hours of self-defense training based on Wen-Do Women’s Self-Defence, which teaches techniques designed to be successful against larger, stronger perpetrators. Unit 4 (relationships and sexuality) includes sex-positive sexual education adapted with permission from the Our Whole Lives program [9,10]. Activities build on what women have learned from the previous 3 units and apply this knowledge to longer-term romantic and sexual relationships. More detail is provided elsewhere [8,11].

Despite its efficacy and availability since June 2016, implementation of EAAA on campuses has been limited, with ongoing and new implementations interrupted during the COVID-19 pandemic. Interest has been high despite the absence of marketing efforts, and demand for training has been strong (15 trainings between 2016 and 2024 for 193 staff), with high satisfaction ratings. Nevertheless, relatively few universities send their staff for training, and the number of campuses implementing the EAAA intervention remains small. Some US administrators have expressed reticence due to the belief that a Canadian RCT is insufficient evidence of efficacy for US students. Resource issues have been an obstacle to the wider scale-up of EAAA despite institutional interest. Local implementation costs roughly US $232 per participant in the first year and US $200 in subsequent years [12]. Only 13 participants need to complete the program for one fewer attempted or completed rape to be experienced [5]. However, on campuses where resources are limited, this cost has been prohibitive. This estimate also does not include the cost and logistical burden of assigning an existing staff member as a Campus Trainer who must be trained and subsequently recruit, hire, train, and supervise a team of near-peer facilitators to offer the intervention on campus. Near-peer facilitators are often graduate students, which generates high turnover among facilitators if they are in 1- or 2-year master’s degree programs, and it can be difficult to recruit facilitators at undergraduate-focused institutions. Adapting EAAA for web-based delivery, which could be delivered to any campus by a centralized pool of peer facilitators, would eliminate the need to have trained staff locally. We estimate that this could reduce intervention costs to an institution by as much as 30%-40%.

In addition to reductions in cost and local implementation burden, a web-based version of EAAA offers key benefits for timing, fidelity, and accessibility of the intervention. Unlike in-person EAAA, students could more easily take the intervention remotely via web-conferencing before arriving on campus or in the preorientation “move in” week, addressing the issue that on many campuses, the highest-risk period for SA is in the first semester [13]. A centralized pool of full-time facilitators would likely enhance intervention fidelity over campus-specific facilitation models, as supervision quality of facilitators at a given institution may vary; further, small institutions where facilitators only offer 1 or 2 sessions per year may not be high enough frequency for facilitators to truly gain expertise with the intervention [14]. Centralization and web-based facilitation should therefore theoretically improve the effectiveness of the intervention across sites. A version of EAAA delivered via the internet would also increase the reach and coverage of the intervention to students at universities where intensive campus-run primary prevention programming is not offered due to a lack of institutional resources, which may disproportionately affect students who attend religious, rural, commuter, tribal, or small campuses.

Our adaptation of EAAA, called Internet-Delivered EAAA (IDEA^3^, pronounced “IDEA Cubed”), retains the synchronous, live-facilitated group learning format over videoconferencing and retains all of the core content of the theory- and evidence-based EAAA. We conducted a rigorous and systematic adaptation for internet delivery using the ADAPT-ITT framework [15]. This is a substantial innovation compared with existing web-based options, which are most commonly click-through or asynchronous trainings (eg, the popular sexual assault prevention for undergraduates training by Vector Solutions; formerly called Haven by Everfi). Asynchronous, individual web-based interventions have failed to reliably change even knowledge or attitudes about SA [16-18] or have had equivocal short-term effects on victimization [19]. Key advantages of the synchronous group format over individual click-through programs include learning from and with peers and peer facilitators, longer duration (at 12 hours) than other web-based programs, and the ability to address comments and questions that emerge from participants, allowing for meaningful integration of the material. Our 2-year adaptation process included extensive consultation with the University of Windsor’s Office of Open Learning staff with expertise in online education, pedagogy, and technology. A theatre test on a small number of first-year students (N=8) led to further revision followed by a single-arm pilot trial (N=65), which provided preliminary evidence of IDEA^3^’s feasibility, acceptability, and effects on the validated intermediary outcomes shown to mediate the effect of in-person EAAA on SA victimization [20]. The pilot study demonstrated the feasibility of the intervention, with strong recruitment and retention. The acceptability of the intervention was high, with participants reporting enjoyment (mean 8.2 out of 10) and willingness to recommend the training to others (mean 8.5 out of 10). Qualitative data from open-ended survey questions and focus groups indicated that within a short follow-up period, many participants had already used information and skills they learned in the program to detect coercive behavior and remove themselves from situations where they felt unsafe. Preliminary indicators of potential efficacy showed that the program increased self-defense self-efficacy and perceived risk of acquaintance rape while reducing acceptance of rape myths and beliefs about female participation in rape, echoing the changes found in these mediators of intervention effect in the in-person EAAA trial [21].

EAAA is part of a broader group of empowerment and resistance interventions developed in the field, but it has the strongest evidence base for efficacy in reducing victimization in North American university contexts. Other high-quality programs for university women had been developed since the late 1980s and were promising, but generally did not work to reduce victimization reliably for survivors or for the longer term [22]. A much longer empowerment self-defense program was found to be effective in reducing sexual violence in a propensity-matched quasi-experiment [23]. Unlike most other interventions in this space, EAAA is effective at reducing victimization in women with and without previous sexual victimization experiences [5,24]. EAAA was developed based on a decade of empirical evidence and theory [25] and, with pedagogical attention to the reality that survivors would always be present in the group, to address the difficult social and emotional context women face in society when the risk of SA is highest from people they should be able to trust (eg, male friends, intimate partners, and other acquaintances) in situations that should be safe (eg, parties, studying with classmates, and dating). EAAA is the only resistance program evaluated in North American universities with a rigorous RCT [4].

We focused on developing a web-based version of EAAA for scale-up, as it is the intervention with the strongest evidence base for yielding immediate, durable, and substantial reductions in assault victimization. While comprehensive prevention of sexual violence will involve ending perpetration and dismantling structural sexism, no existing interventions have been rigorously shown to durably reduce campus sexual violence perpetration, and a cultural shift is a long-term effort. In the meantime, EAAA prevents 1 attempted or completed rape for every 13 women who take the program, while reducing self-blame in the event of an assault [5,24]. An adaptation of EAAA for internet delivery would prevent imminent threats to safety and reduce sexual violence for women now and at scale, as work to end perpetration continues [26].

The next step is an RCT whose aim is to test whether IDEA^3^, when compared against a brief control workshop (“usual care,” ie, consent education), can deliver reductions in SA victimization of sufficient size to warrant scale-up to campuses in North America. Given that web-based interventions sometimes fail to produce benefits as strong as in-person ones [27], we have powered our trial to detect an intervention effect that is 80% as large as the effect size of in-person EAAA. We hypothesize that IDEA^3^ will reduce the 1-year incidence of completed rape by 37.75% (absolute difference of 3.7%) among first- and second-year undergraduate women in the United States, as compared with a consent workshop control condition. If successful, this trial of a web-based adaptation of the only intervention proven to prevent campus sexual violence and sustain those benefits in the longer term has the potential to revolutionize the fight against campus SA.

## Methods

### Study Design

This is a multisite, open-label, RCT.

### Inclusion and Exclusion Criteria

A total of 1920 first- and second-year undergraduate students will be enrolled in Canada and the United States. To be eligible, participants must (1) identify as women, (2) be between the ages of 17 and 24 years, (3) be first- or second-year undergraduate student, (4) be available during one of the intervention schedule blocks, (5) provide the contact information for another woman undergraduate who they would be willing to participate in the intervention with or express willingness to being partnered with another woman undergraduate at their university, (6) have access to internet and be willing to use the videoconferencing software ZoomPro (Zoom Video Communications, Inc) for the intervention, (7) provide informed consent, including to be recorded while participating, and (8) complete the baseline survey and be randomized with a partner who also meets all the criteria. At one site (University of Central Florida), participants must be 18-24 due to state law. Participants whose partners drop out within 24 hours of the first intervention session or do not show up for their assigned intervention after randomization will be permitted to participate alone. There are no exclusion criteria.

### Recruitment

Recruitment began on October 2, 2023, at 4 sites: the Universities of Windsor and Guelph in Canada and the Universities of Michigan and Nebraska-Lincoln in the United States. In year 2 of the trial, recruitment was ended at the University of Nebraska and paused at the University of Michigan due to the departure of the site principal investigators (PIs) from those institutions. In year 3 of the trial, recruitment is planned at the University of Guelph, the University of Michigan, the University of Maryland, and the University of Central Florida. The 6 total sites represent diverse students, sizes, and contexts (eg, rural vs urban, proportion of students in residence, racial, and geographic diversity). Recruitment will only take place during the academic year when classes are in session. Multiple methods are used to attract participants to the study, including (1) recruitment emails through the registrar, faculties, or departments to all eligible students, (2) research participant pools, (3) social media ads or posts to relevant Facebook groups (eg, groups for students at a particular university), and (4) flyers will be distributed at on-campus events, posted in women’s bathrooms on campus, and sent digitally to student newsletters/newspapers, student organizations, or student-specific listserves at the sites of Universities. Not all recruitment methods will be used at all sites (eg, emails are permitted through the Registrar at certain sites but not others).

### Prescreening Survey

Links or QR codes in advertising materials (emails, flyers, and ads) take potential participants to a website that has the semester’s updated survey link or directly to the prescreening survey in REDCap (Research Electronic Data Capture; Vanderbilt University) to determine eligibility. The screener begins with a brief description of the study, followed by the screening survey.

Participants give their initial consent to provide demographic information in the screener to determine their eligibility for this research. Demographic information provided (eg, sexual identity and race/ethnicity) also allows us to see whether recruitment strategies are working to reach diverse students. If participants (1) do not consent to provide demographic information or (2) are ineligible based on the screening criteria, they are sent to a page thanking them for their interest.

Participants attend the IDEA^3^ program in pairs for support and in order to adequately practice and learn the self-defense tactics in the third unit with a partner. Using the same protocol we used in our pilot trial [20], after completing the screening survey, eligible participants are sent to an additional page where they are asked a series of questions about the partner with whom they would be willing to participate. Partners must meet study eligibility criteria. Students can either participate with a friend or consent to be partnered with another undergraduate woman by the study staff. Potential participants must also indicate which one of the intervention session blocks scheduled for that semester they are available for. Although the control arm intervention condition only meets once (see below), participants must state they are available for the 4 dates allotted for the IDEA^3^ program in case they are assigned to the intervention arm of the trial.

Students who wish to participate with a friend are asked to provide their own contact information and the first and last name of their friend. If they are willing to provide the friend’s email address, they can personalize an email invitation sent directly through REDCap, or if they prefer to communicate with their friend first, we send them an email they can forward to that friend with a link for them to sign up. If that friend is not interested, students have the option of proceeding in the study with a match done by the research team or discontinuing their involvement. If they believe their friend has already signed up for the study, they can indicate this and, if verified, that person is contacted to ensure they are willing to be matched with the student. Participants who do not provide the name for a potential program partner and are unwilling to be partnered with another student by study staff are sent to a page thanking them for their interest, as they are not eligible for the study. Referred program partners progress through a similar prescreening process via an emailed REDCap link. Once both partners have completed the prescreening survey and have screened as eligible for the study, they are “matched” and their ID numbers are linked in REDCap.

Partnered participants are introduced (if they were not already friends) by email and invited to get in touch to discuss where they will get together to participate. Further, they are provided with a link where they can schedule their individual screening interview or request further information.

### Screening Interview and Informed Consent

Interested students who meet the initial prescreening criteria and have been successfully matched with a partner receive an email with a link to book a 30-minute screening interview using Calendly. Up to 5 reminder emails/texts are sent over a 10-day interval until the student schedules an interview or is presumed to be no longer interested in the study. When potential participants book, they are sent a confirmation of their interview time and date, a Zoom link with instructions on how to use Zoom, and the full consent form to review before the interview. The main points from the consent form are reviewed aloud with the participant by the study staff, and questions are answered. Participants provide consent verbally at the start of the scheduled interview (or, if not given, participants are thanked, and the interview is ended). The screening interview is conducted by an intervention facilitator and potential participants are required to turn on their camera for the interview as (1) this allows additional screening for age and gender eligibility and (2) participants are required to come to intervention sessions with cameras on, and using a camera during the screening interview confirms they have a working camera that they can use during intervention sessions.

Facilitators review the format of the intervention with participants to make sure the participant is willing and able to meet in person with the partner and that they have a suitable and safe space to participate together. Facilitators assess any physical limitations that might affect participation in the self-defense portion of the program (to inform adaptations for accessibility if they are assigned to the intervention arm), ensure participants feel comfortable using Zoom, and ensure participants have a stable Wi-Fi connection. They are asked if they are willing to sign up for a GroupMe account and be included in group messages to other participants assigned to their intervention block using this app. GroupMe is a messaging app designed for multi-person chats. Facilitators use this app to send messages to participants in the IDEA^3^ arm (after they come to the first session and learn their assignment), about upcoming program sessions, resources, etc. Participants do not have to have a GroupMe account to receive these messages, but they need one to chat with other participants. For participants who have requested to be partnered with someone they do not know, this interview allows them to discuss any concerns/reservations about this process and go over where they need to meet their partner before their first or only program session. Both partners in each participant pair will be required to individually complete this one-on-one interview and provide informed consent before being allowed to enroll in the study.

### Randomization

When participants have completed their screening interview, they receive an email inviting them to complete the baseline survey (see section below on assessments for more details). The baseline survey must be completed no less than 24 hours before their first program session (or only program session for control participants). The email contains an individualized URL taking them directly to this first survey, which takes 30-45 minutes.

Randomization of the pair will occur once both participants in the pair complete their screening interview and baseline survey, and no later than 24 hours before the first (or only) session. If one member of the pair fails to complete the baseline survey, the other member of the pair will be given the choice of continuing in the study with a different partner (time permitting), rescheduling for a later intervention session, or discontinuing the research. In rare circumstances, that is, if the participant has been re-paired once or more, there is insufficient time to re-pair, or no one else is available for a new pairing, she will be individually randomized and permitted to complete the intervention by herself. Once the pair is randomized, they are sent a Zoom link for the session through REDCap. This link does not identify their random assignment. The facilitators/research assistant (RA) reveal the assignment once everyone is present in the Zoom room at the scheduled time.

The trial biostatistician is responsible for setting up the randomization protocols in REDCap. Randomization is stratified by site using permuted block sizes of 2 and 4, and participant pairs are randomized 1:1 to the IDEA^3^ program or control. Randomization occurs after baseline completion so that participation in the trial cannot be affected by knowledge of intervention assignment. The site coordinators generating the random assignment for a participant pair are not able to view the random allocation sequence.

### Interventions

#### Both Arms

After providing informed consent in the screening interview and before the first (or only) session, a Program Resource Kit is delivered to participants by mail (if time permits) or hand-delivered by study staff. Kits include some sealed envelopes (marked unit 1-4) with resources necessary for both the control and intervention arms, with instructions not to open the envelopes until they are instructed to do so at their first intervention session. Kits also include some snacks and small “swag” items. Both arms receive identical kits. Receiving gifts and snacks before the first session also encourages attendance at the first session.

#### Intervention Condition

The IDEA^3^ SA resistance program follows the same structure as EAAA, with four 3-hour units led by 2 near peer-facilitators with content designed to help women more quickly and effectively recognize and resist coercion (see the *Introduction* section for more detail). IDEA^3^ retains the interactive modality of EAAA by using games, discussions, and other activities.

This intervention is presented by a pair of highly trained woman-identified “near-peer” program facilitators (ie, a few years older than the participants). Unlike the original in-person intervention, a tech facilitator is present at all sessions to facilitate the Zoom technology, troubleshoot participant technical difficulties, assign breakout rooms, provide participants with links to external games/group apps, record sessions, and perform other technical duties before, during, and after each session. The presence of the tech facilitator allows the program facilitators to focus on content delivery and facilitating participant discussion and engagement.

Participants receive the intervention in pairs via Zoom in a private room with enough floor space to practice basic self-defense techniques, and where they know they will not be disturbed. If a pair of participants does not have an appropriate room available to them, a university-sponsored space for each pair is arranged. These Zoom sessions include a minimum of 1 and a maximum of 8 pairs of participants (16 women total) taking part in the intervention together.

#### Control Condition

Participant pairs assigned to the “usual care” control arm will receive one 60-minute session. The workshop focuses on (1) defining consent, including what components need to be present for consent to be meaningful, and (2) expanding the ways in which participants feel comfortable giving, refusing, and asking for consent. The control condition was selected to mimic what is currently available, “usual care” on typical college campuses, which is a brief consent workshop. The content is therefore based on the common components of consent workshops held at the study sites and community-based consent workshops (confirmed with participating campus materials and in consultation with the Ontario Coalition of Rape Crisis Centres).

As with the intervention arm condition, participants receive the consent workshop in pairs via Zoom in a private room (their own or provided to them on campus). These Zoom sessions include up to 8 pairs of participants (16 max) taking part in the workshop together. The control workshop is given by a well-trained RA. Participants who are assigned to the control arm will be informed of the importance of the control group to the study and their ongoing participation. They are made aware that they will be contacted in a year and offered the opportunity to take the full IDEA^3^ program.

### Training of Facilitators and Research Assistants Conducting Interventions

IDEA^3^ has been manualized for program facilitators and tech facilitators and, as is the case for the in-person version of the program, includes scripts, instructions on how to conduct activities, and troubleshooting advice. An EAAA lead trainer delivers the theory and program content-based 5-day facilitator training. Training also includes a certified Wen-Do Women’s Self-Defence instructor leading an in-person 15-hour Wen-Do Women’s Self-Defence Basic Course and another day of instruction in how to safely teach the self-defense content remotely over videoconferencing. Tech facilitators attend a portion of this training and have additional training in their tasks. Program facilitators and tech facilitators receive additional training to perform their research tasks (eg, screening interviews and tracking attendance in REDCap) from PIs. RAs are trained to conduct the control intervention by PIs. Research training of Site/Country Coordinators and RAs is conducted by the Trial Project Manager and PIs.

### Feasibility/Acceptability

#### Postsession Evaluations

At the end of each IDEA^3^ session and at the end of the control arm workshop, participants are given a link to an anonymous 5-minute REDCap survey with closed- and open-ended questions on their evaluation of the session to assess feasibility and acceptability, and allow the chance to offer feedback on the program. Participants are asked to complete the survey immediately to minimize recall bias, and to increase response rates, participants only receive entries into the two $25 raffles for the session if they fill out the evaluation form.

#### Attendance/Dose and Fidelity

Attendance at the assigned intervention/control sessions will be recorded by the tech facilitator/RA to allow us to examine the effects of dose and attrition. All sessions in both arms will be recorded, and a randomly selected 25% of these recordings will be assessed for facilitators’/RAs’ fidelity to the protocols for that arm of the trial. Consent for video recording of each program session is a requirement of participation in the research, is included in the Consent Form, and is discussed in the screening interviews.

### Outcome Assessments

Web-based surveys will be administered to participants at 4 time-points as explained in the following subsections.

#### Baseline Survey

After completing the screening interview and giving consent, participants are invited to complete the baseline survey. The survey includes a number of demographic questions (eg, year of study, age, ethnicity, sex assigned at birth, gender identity, sexual identity, sexual orientation, current relationship status, number of sexual partners, and age of first sexual contact), primary outcome assessment (Sexual Experiences Survey-Short Form Victimization [SES-SFV]) [28], secondary outcomes scheduled for the baseline (Self-defense Self-efficacy Scale) [29], perceived risk for acquaintance SA [30] (1 item), the Perceived Causes of Rape Scale [31], and the Updated Illinois Rape Myth Acceptance Scale [32], and tertiary outcomes (Acceptance of Sexualized Aggression Scale [33], 13 items from the Sexual and Reproductive Empowerment Scale [34]).

#### Postintervention Survey

One week after the completion of the final session for participants in the intervention arm, participants in the same recruitment cohort in both arms of the trial will be invited by email to complete the postintervention survey via the web-based REDCap platform. This survey includes the same measures used in the baseline, excluding demographic questions. It includes additional secondary outcome measures: (1) a measure of Risk Assessment and Resistance developed by Testa et al [35] built on items from Norris et al [36] and Davis et al [37], and (2) an open-ended question measuring knowledge of effective resistance strategies and willingness to use them if a man they knew tried to force them to have sex when they did not want to. It also contains “contamination questions” to determine whether and to what extent participants in the intervention arm shared information with those in the control arm, or control arm participants had IDEA^3^ content shared with them. Participants who indicate on the postintervention SES-SFV that they experienced a completed or attempted rape will also be asked follow-up questions relating to the first (and the most recent if more than one) SA (eg, the date the assault happened, her relationship to the perpetrator, etc) and these same participants will also be asked to complete the Rape Attribution Scale [38] to assess self-blame.

#### 6- and 12-Month Follow-Up Surveys

Participants will be invited to complete the 6- and 12-month follow-up surveys approximately 6 and 12 months after completing their baseline survey. The timing is adjusted to avoid the summer months when response rates are much lower (based on the research team’s previous research). These surveys will consist of the same measures used in the postintervention survey, except that the risk assessment and resistance measure is replaced with 2 different versions of a risk perception task [39], one at each time period, as the risk assessment and resistance measure is not designed for repeat use.

#### All Surveys

At each follow-up point, participants will receive an email invitation through REDCap to complete each of the surveys. A combination of text and email reminders (up to 5) to complete each survey will be sent every 24-48 hours to any participants who have not yet completed them. This is fewer than Dillman et al’s [40] recommended number (7 reminders), but we have found 5 sufficient to maximize survey completion rates. Participants are reminded of the study structure and reconsented at 6- and 12-month time points via an introductory page to the survey before progressing to the questions.

At the end of the postintervention and 6-month surveys, participants are asked to provide updated, detailed contact information (primary and secondary cell phone numbers and email addresses) so that we can contact them for subsequent follow-ups. For use in the event that an email or text is unable to be delivered, we will ask for (1) permission to contact the Registrar’s Office in case the contact information they have provided us is out of date and (2) contact information for 2 people who would know how to get in touch with them in case they have not updated their contact information with the Registrar. If text messages or emails are undeliverable at the 6- or 12-month surveys, we will ask the Registrar or the provided contacts for updated contact information only for those participants who have given us permission to do this. If we reach out to the participants’ nominated contacts, we will not share any information about the research study when we do so. We will only ask these individuals to ask the participant to contact us for those participants who have given us permission to do this.

### Incentives

In the second year of recruitment, the incentive for the 20- to 30-minute screening interview was increased from $5 to $10, based on attrition between preliminary screening/matching and the interviews. This change is reflected in the description of incentives that follows. For participation in this research, participants receive incentives (preloaded gift cards) of up to $105 or receive up to $70 and 2.5 course bonus points for those recruited from Participant Pools. Specifically, nonpool participants receive $35 for completing the screening interview, baseline, and 1-week postintervention surveys ($20 for screening and baseline alone if they do not complete both surveys), $30 for the 6-month survey, and $40 for the 12-month surveys. Pool participants receive 2.5 bonus points for completing the screening interview, baseline, and postintervention surveys (combined) which occur in the same semester in which they are registered for the participant pool (1.5 pts if they do not complete both surveys), and the same monetary incentives as nonpool participants for the remaining 2 follow-up surveys (which occur outside of that semester).

For each session attended and postsession evaluation completed, participants (in both trial arms) are entered into a postsession raffle for two $25 draws among the people who attended their session that day (max 16 participants). Participants assigned to IDEA^3^ are therefore eligible for up to $100 in session-end raffles (depending on how many sessions they attend) compared with $25 for control group participants, to compensate them for the longer time commitment involved. Further, for each intervention session attended, all participants (in both trial arms) receive a ballot for an end-of-term $250 raffle at each site.

Increasing incentives for each survey completed encourage students to stay in the trial for its full duration after the intervention is complete and maximizes response rates for follow-up surveys within a range supported by the published literature [41], our previous RCT [5], and our implementation trial [6]. The use of various incentives is standard in psychological research and, increasingly, in clinical trials, providing the size of the payment does not put “undue” pressure on individuals to participate, and the sample is not drawn from a “vulnerable population” [42,43]. Our survey incentives ($105 over the course of multiple surveys for a year) are well within the range considered acceptable (adjusted for inflation) by authors publishing in Contemporary Clinical Trials [44].

Every intervention arm participant who completes all 4 sessions of the IDEA^3^ program will receive a certificate of attendance for their resumé.

### Outcome Measures

#### Primary Outcomes

The primary aim of the trial is to test whether IDEA^3^ can reduce the 1-year incidence of completed rape among women attending university in Canada and the United States. Completed rape will have occurred when a participant indicates she has had at least one experience of threatened, forced, or drugged completed (not attempted) sexual activity (oral, anal, or vaginal intercourse)—answered “once” or more to any of 9 items (2c-e, 3c-e, and 4c-e) in the period between the last outcome measurement and the current day (SES-SFV). Dates of occurrences will be recorded using a calendar function to allow time-to-event analyses. The SES-SFV has been used in very large university and college and community samples and is considered the gold standard. We use the gender-specific version (male perpetrator) due to the focus of the intervention. Responses on the scale are stable across administrations [28] and are comparable to those received through interviews [45]. The validity of the scale has been demonstrated in many published studies [28,47]. Predictive validity has been demonstrated with correlations between sexual victimization and variables predicted to be influenced by it, such as trauma symptoms [28].

#### Secondary Outcomes

The secondary aim of the trial is to test the hypotheses that IDEA^3^ can reduce the 1-year incidence of: (1) attempted rape; (2) attempted or completed sexual coercion; and (3) forced sexual contact (nonpenetrative SA). Attempted rape will have occurred when a participant answered she has had at least one experience of attempted, threatened, forced, or drugged completed sexual activity (oral, anal, or vaginal intercourse)—answered “once” or more to any of 9 items on the SES-SFV (5c-e, 6c-e, 7c-e) in the same period. Sexual coercion will have occurred when a participant reports one or more incidents of verbally coerced (excluding threats of physical harm) nonconsensual oral, vaginal, or anal intercourse—answered “once” or more to any of 6 items (2a-b, 3a-b, 4a-b). Attempted sexual coercion is assessed when a participant reports that sexual coercion was attempted but did not occur—answered “once” or more any of 6 items (5a-b, 6a-b, 7a-b). Forced sexual contact will have occurred when a participant indicates she has had an experience of threatened, forced, or drug-facilitated sexual touch (excluding intercourse)—answered “once” or more to any of 3 items (1c-e). Dates of occurrences for the first reports in any period will be recorded using a calendar function to allow time-to-event analyses.

#### Tertiary Outcomes

The main tertiary aim is to test the hypothesis that IDEA^3^ can positively affect the 6 outcomes shown to be mediators of decreases in SA victimization [47]: (1) increase women’s ability to detect risk in hypothetical situations, (2) increase women’s knowledge of and willingness to use the most effective self-defense strategies, (3) increase women’s confidence that they could defend themselves if confronted with a SA situation, (4) increase women’s perception of their own risk of SA by male acquaintances, (5 and 6) reduce rape myth beliefs and woman-blame that hamper risk detection.

The expanded cognitive appraisal model [48] upon which the intervention is built proposed a process through which changes to the primary outcome of rape victimization would be accomplished. Chained mediation analysis of the original RCT data supported this theory and showed that 3 measures (risk detection, knowledge, and willingness to use effective strategies, and self-defense self-efficacy) were primary mediators that explained 95% of the decrease in completed rape and 76% of the decrease in attempted rape [47]. Other attitudes and beliefs (perceived risk of one’s own SA risk from male acquaintances, general rape myth beliefs, and belief in female precipitation of rape) were more distal mediators that led to improvements in the primary mediators. Measurement of mediators (common to the original EAAA RCT) will allow targeted revision to the IDEA^3^ program if necessary to strengthen program material meant to impact the mediators that were not significantly affected by the intervention.

Tertiary aims additionally include testing the hypothesis that IDEA^3^ can, (7) reduce women’s self-blame if a rape occurs, as previously demonstrated with the in-person version of EAAA, as well as exploratory hypotheses related to whether IDEA^3^ can, (8) reduce attitudes related to acceptance of sexualized violence using the Acceptance of Sexualized Aggression scale [33] and, (9) increase sexual empowerment, using 13 items drawn from the Sexual and Reproductive Empowerment Scale [34].

### Methods for Protecting Against Sources of Bias

#### Selection Bias

Randomizing a large number of participants is expected to minimize selection bias, that is, minimize baseline differences between the intervention and control groups. Selection bias is further minimized by concealing the random assignment to participants until they complete the baseline assessment and arrive at the virtual intervention session.

#### Outcome Assessment Bias

Blinding is not possible in this trial. However, bias in outcome assessment is minimized by having the primary outcome, SA, collected with the best measure of coercive sexual experiences available (SES-SFV) [45]. This measure will be applied equally to all study participants. The measure has been tested on tens of thousands of undergraduates, and it has high reliability and validity [28,45]. Self-report through the SES-SFV has been the gold standard for assessing SA experience, as external adjudication is not possible and allows comparison to the original RCT. The instrument follows best practice for not using terms like “sexual assault” directly but instead assessing by describing the behavior (eg, “A man put his penis into my vagina, or inserted fingers or objects without my consent by using force, for example, holding me down with his body weight, pinning my arms, or having a weapon”). This measure, therefore, avoids issues with survivors not labeling their experience as “assault” despite it meeting the definition as intended.

#### Differential Dropout

Our pilot testing prior to the original EAAA RCT indicated that dropouts are minimized by having follow-up times coincide with the school year rather than with holiday breaks. We use the same follow-up timing in this trial. As we have done previously, we use frequent reminders (sent out by RAs at each site and automated within REDCap) for those who have not responded to maximize response. For students who have not completed a 6- or 12-month follow-up survey within 2 weeks after the final reminder, we invite them to complete a shorter survey that just includes the SES-SFV (primary and secondary outcomes) at 6 months and 12 months. Taken together, these methods reduce the attrition rate considerably. There is a possibility that women in the control arm may not feel as connected to the research and drop out of the study as a result. We have reduced the likelihood of differential dropout by providing a brief intervention (consent workshop) rather than using a no-contact control, a participant kit including snacks and resources mailed to the home of control participants as well, increasing incentives and personalized email/text contact, as well as offering the opportunity for control participants to receive IDEA^3^ at the end of the trial. Recruitment materials present both intervention and control programs as valuable to participants. As evidenced by our past research, these maximize the connection to the trial to reduce or eliminate differential attrition.

#### Between-Group Contamination

While our own and others’ research show that significant contamination is not likely to occur for an intervention of this length [49], as we have done previously, we will ask all participants at 1-week, 6- and 12-month follow-up the following questions: “Do you have any friends who were assigned to the other program in this study?” If the answer is “yes,” they are asked, “Did she/they share anything with you about what they learned in their program?” or “Did you share anything that you learned in your workshop with her/them?” depending on their group assignment. An open text box asks them to enter what was shared. We will describe the amount of contamination occurring and discuss its possible influence on the findings.

#### Noncompliance and Loss to Follow-Up

Noncompliance (aka “no show”) is defined as not attending the assigned intervention after randomization. Lost to follow-up is defined as not being able to ascertain participants’ SA status at 1 year.

To minimize attrition, participants’ sessions will start close to the beginning of the term or closely following midterms (rather than during final assignment and examination season when participants have competing priorities). We will use incentives, reminder emails, and texts 1 week and 1 day prior to the first session, and reminder texts by facilitators for those in the IDEA^3^ arm for subsequent sessions. Using a similar level of incentives, we attained full EAAA attendance from more than 78% of participants in the previous RCT and over 90% in the IDEA^3^ pilot trial.

Nonresponse on follow-up surveys will be defined as refusing to complete one or more follow-up surveys. We use a number of procedures known to maximize response rates for follow-up surveys [40], such as confirmation of contact information at each follow-up and multiple reminder emails/texts. We use increasing incentives ($10 baseline, $15 1-week, $30 6-month, $40 12-month surveys) within a range supported by the published literature [41] and our previous RCT [5] and implementation trial [6] to maximize response rates. We have found that web-based surveys (with all appropriate security and privacy measures taken) improve the ease with which women can participate. The follow-up outcome surveys take less than 30 minutes to complete, which maximizes response rates [40].

### Sample Size Considerations

In calculating the sample size for the proposed trial, we used estimates of the 1-year incidences and difference in completed rape from the previous trial (9.8% control vs 5.2% EAAA=4.6% difference [5]). However, we conservatively powered the trial so we can still detect meaningful differences even if IDEA^3^ is less efficacious than in-person EAAA, that is, it achieves 80% of the previously observed difference (80% of 4.6%=3.7%). Therefore, assuming 1-year incidences of completed rape in the proposed trial of 9.8% in the control group versus 6.1% in the intervention group, a sample size of 1676 will have 80% power to detect an absolute difference of 3.7% (relative risk reduction of 37.75%) at a 2-sided 5% level of significance. As participants in the IDEA^3^ sessions will be in pairs, the sample size is further inflated by a design effect of 1.031, based on a within-pair correlation of 0.031 from the previous trial. Including an estimated maximum 10% lost-to-follow-up, a total of 1920 participants will be enrolled in the trial (1920=1676×1.031/0.90). Using estimates for the secondary outcomes from the previous trial [5] and similarly, assuming 80% efficacy compared with the in-person intervention, the proposed sample size yields 97% power for comparing 1-year differences in attempted rape (9.3% vs 4.6%), 92% power for sexual coercion (22.6% vs 16.1%), and 99% power for sexual contact (39.1% vs 28.5%). Using estimates for the tertiary outcomes from the previous trial [21] and similarly, assuming 80% less efficacy, the proposed sample size yields > 99% power for comparing 1-week postintervention group differences in risk perception (Cohen *d* 0.58), rape myths (Cohen *d*=0.48), risk detection (Cohen *d*=0.54), and self-defense self-efficacy (Cohen *d*=0.64).

### Statistical Analyses

Analyses will be performed after all participants have completed their 1-year follow-up survey. Following a modified intention-to-treat principle, a participant will be included in the analyses if they are randomized and complete one or more postrandomization surveys. The analyses will compare the incidence (ie, first occurrence) of each primary and secondary outcome between the IDEA^3^ and control groups with Kaplan-Meier failure curves (indicating the cumulative percentage of first outcomes among women in the respective groups) and log-rank tests. Participants who are lost to follow-up or drop out will have their data censored at the time of last contact. To account for the correlation between the paired observations, variance estimates will be appropriately inflated for within-pair clustering with the use of estimates of the design effect [50]. In case of baseline imbalances in participant characteristics, Cox proportional hazards regression models with a robust sandwich covariance matrix to account for the clustering will be used to compare the IDEA^3^ and control groups while controlling for the confounding factors. The literature [24,51,52] suggests that prior SA can be a modifier with respect to the effectiveness of SA resistance interventions. Therefore, intervention effects will be compared for women with and without SA histories at baseline by adding a cross-product term to the Cox regression model and testing for statistical significance. For the tertiary outcomes, which are all continuous, linear mixed models will be used to analyze the data that mirror a multilevel repeated-measures study design. A random intercept will be included in the models to account for the correlation between the paired observations, and a first-order autoregressive covariance structure will be used to characterize the interdependence of the repeated measures over time. A cross-product term between group and time will also be included in the models. Participants who are lost to follow-up or drop out can affect the validity of the group comparison if (1) the outcome is related to being lost or dropped, and (2) it is differential between groups. Therefore, baseline characteristics of women who are lost and drop out will be compared with women who complete the trial. In addition, the missing data mechanism will be formally tested [53] to determine whether it is Missing Completely at Random or missing at random. For completeness in assessing the effect of missingness, 2 additional analyses will be conducted: (1) a “per-protocol” analysis among participants who attend all sessions and respond to the 1-year SES-SFV questions; (2) multiple imputation of 5 data sets using a discriminant function for binary outcomes and Markov chain Monte Carlo for continuous outcomes, assuming the missing data mechanism is missing at random.

### Ethical Considerations

Ethical approval for the trial was received from the University of Windsor Research Ethics Board (reference number 42876). Other university sites (eg, University of Guelph, University of Michigan, University of Maryland, University of Nebraska-Lincoln, and University of Central Florida) ceded ethical review to the Windsor Research Ethics Board.

Our ethics and safety protocols have been developed and refined over many pilot studies and 2 trials. There are limited risks related to the content of the intervention and the survey questions on SA. Women know the topic of the intervention and the study prior to volunteering and therefore can evaluate their readiness to participate. Informed consent is fully voluntary, and participants can opt out or leave the study at any time. Participants provide verbal informed consent in the screening interview, which is documented by study staff. Brief emotional discomfort may still occur for participants, particularly if they have an assault history. Session evaluations demonstrate that survivors rate participation in EAAA positively, and our own [25] and others’ [54,55] research confirms that survivors perceive research on these topics as beneficial and important. The self-defense instruction adds a limited physical risk. Risks are minimized through protocols associated with the research (eg, internet and local resources and referral lists provided at every time point), intervention (eg, trauma-informed intervention content and process, screening interviews to assess physical safety of space and participants’ physical limitations, clear safety instructions for self-defense practice, extra-large external monitors for clear view of participants to monitor their safety), and training (eg, extensive facilitator training including on dealing with disclosures, providing referrals, and monitoring the safety of self-defense practice). None of the women participating in the original RCT (N=893) or implementation effectiveness trial (N=603) reported adverse events. Consultation with Deb Chard, Senior Wen-Do Women’s Self-Defense instructor, during the adaptation process enhanced our facilitator training and participant safety monitoring for the virtual environment and the theatre, and a pilot trial of IDEA^3^ confirmed their efficacy.

Participants receive up to $105 for study activities, with the possibility of winning additional raffle incentives. Identifiable data are stored on a secure REDCap database at the University of Windsor. All survey data will be reported in the aggregate and deidentified; identifiable data will be deleted at the end of the study after all the data are cleaned and merged across time points. Video recordings of the screening interviews will be deleted at the end of the semester, and video recordings of the intervention sessions will be deleted once the project is over and all fidelity ratings have been completed.

### Steering Committee and Trial Oversight

The Steering Committee includes the PIs and 4 coinvestigators. Our psychosocial educational interventions do not require a data safety monitoring committee. Research decisions for all sites are made by the trial project manager, and weekly communication with the Site Coordinators during active recruitment and intervention periods will ensure consistency on trial issues. Integrity of the trial at each site is the responsibility of the Site Coordinators. The trial project manager is supervised by the PIs; the site coordinators are supervised by the site investigators. Weekly virtual meetings are held between the trial project manager, site coordinators, PIs, and coinvestigators during recruitment semesters, and meetings between the trial manager and all investigators (virtually) monthly and (in-person) annually.

## Results

This trial was funded in October 2022. Recruitment began on October 2, 2023. The first participant pair was randomized on October 23, 2023. A total of 683 participants had been enrolled in the ongoing trial by August 2025. Recruitment will end by May 2026, and data collection will end in May 2027. Results are expected to be published in 2028.

## Discussion

### Key Findings

Findings from this trial will determine if IDEA^3^ can successfully reduce sexual violence against undergraduate women as compared with a consent workshop control condition. In addition to its unique contribution to the published literature as what may be the first synchronous web-based program shown in a randomized trial to reduce SA victimization, the results of the trial will be used to (1) produce a maximally effective manualized IDEA^3^ intervention manual and facilitator training protocol for implementing the intervention in universities across North America or (2) provide direction for further research into which aspects of the intervention need to be strengthened before it can be widely disseminated. The data will also be used to indicate how long the effects of the intervention last and whether refresher sessions are necessary.

Findings from the trial will be disseminated in peer-reviewed publications as well as products such as briefs and infosheets designed for campus decision makers and students.

### Strengths and Limitations

Strengths of the trial include the randomized design against an active control, use of validated measures for outcome assessment, a 1-year follow-up period, and the use of multiple sites in 2 countries to enhance external validity. Limitations of this trial include the restriction of recruitment sites to 2 Canadian (both in Ontario) and 4 US campuses that are all large public institutions, which may limit generalizability. However, campuses were thoughtfully selected for diversity in geographic regions of the United States (Midwest, mid-Atlantic, and Southern) and class and racial diversity. While a 12-month follow-up period is meaningful and beyond what is typical for more campus SA prevention intervention evaluations [19,56], a 2-year follow-up period would allow for more assessment of the durability of intervention effects.

### Future Work

The results of this trial will also be used to provide a foundation for further adaptation work for high-risk minority populations with unique vulnerabilities for SA. If the intervention is effective, we will use the intervention as the foundation for our work to adapt IDEA^3^ for trans and other gender-diverse students, where the smaller proportion of identifying students within one campus population would make the web-based intervention more feasible than an in-person version at every university. As compared with in-person EAAA, where implementation was in the hands of a specific university for its students alone, IDEA^3^ may also provide an opportunity for wider dissemination to young women aged 17-24 years who are not enrolled in or even living near a university and who are at as high or higher risk of SA [57].

## Abbreviations

EAAA: Enhanced Assess, Acknowledge, Act
IDEA^3^: Internet-Delivered EAAA
PI: principal investigator
REDCap: Research Electronic Data Capture
RCT: randomized controlled trial
RA: research assistant
SA: sexual assault
SES-SFV: Sexual Experiences Survey-Short Form Victimization

## Data Availability

Data will be made available upon reasonable request from the authors.
